# V-Shaped Incision of the Proximal Cartilage for High-Caliber Mismatch in Bronchoplasty

**DOI:** 10.1016/j.atssr.2024.04.006

**Published:** 2024-04-24

**Authors:** Yukio Watanabe, Aritoshi Hattori, Mariko Fukui, Takeshi Matsunaga, Kazuya Takamochi, Hisashi Tomita, Kenji Suzuki

**Affiliations:** 1 Department of General Thoracic Surgery, Juntendo University School of Medicine, Tokyo, Japan

## Abstract

**Background:**

The problem of high-caliber mismatch in bronchoplasty is not uncommon. This report describes a technique using a V-shaped proximal cartilage incision to adjust high-caliber mismatch.

**Methods:**

Among 255 patients who underwent tracheoplasty or bronchoplasty at a single institution (Juntendo University School of Medicine, Tokyo, Japan) between February 2008 and December 2022, 12 patients (4.7%) who underwent bronchoplasty with a proximal cartilage V-shaped incision for the adjustment of high-caliber mismatch were investigated. Bronchial anastomosis was performed using a continuous running monofilament suture at the bottom of the cartilage. Interrupted 3-0 or 4-0 monofilament sutures were used for the remaining cartilaginous tissue. Before completing the cartilaginous suture, a V-shaped incision was made in the proximal cartilage at the junction of the membranous portion. The cartilage and membranous portion of the incision were sutured using 3 interrupted sutures with 4-0 polydioxanone sutures. Finally, the membranous portion was sutured to complete the anastomosis.

**Results:**

Eleven patients were men, and the median age was 66 years. The histologic diagnoses were adenocarcinoma in 2 patients and squamous cell carcinoma in 10 patients. Three patients underwent operation after definitive chemoradiotherapy. Right sleeve pneumonectomy, right upper sleeve lobectomy, type A extended-sleeve lobectomy, and type C extended-sleeve lobectomy were performed in 7 patients, 3 patients, 1 patient, and 1 patient, respectively. No anastomotic complications were observed. The V-shaped incision group had a significantly higher frequency of right sleeve pneumonectomy than the group without the V-shaped incision (*P* < .01).

**Conclusions:**

Creation of a proximal cartilaginous V-shaped incision is a useful technique for adjusting high-caliber mismatch, especially in right sleeve pneumonectomy.

## Visual Abstract

**Figure:**
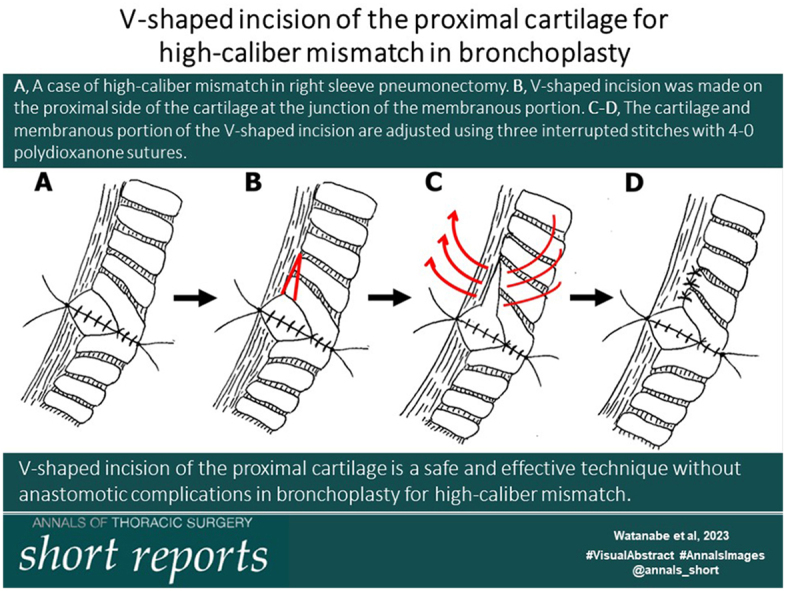

**In Short**

▪ The proximal side of the cartilaginous V-shaped incision is a useful technique for adjusting high-caliber mismatch, especially in right sleeve pneumonectomy.
▪ The key to releasing the tension is to make a V-shaped incision in the cartilage longitudinally relative to the short-axis incision length.

Central-type lung cancer sometimes invades the main bronchus, which requires sleeve pneumonectomy or sleeve lobectomy to achieve complete resection. We sometimes encounter the problem of high-caliber mismatch during bronchoplasty. The large size discrepancy between the proximal and distal stumps requires not only an appropriate anastomotic technique but also an appropriate approach to the stump. The methods reported for adjusting high-caliber mismatch in bronchoplasty include a couple of adjusting sutures in the membranous part of the larger stump of the anastomosis,^1^^,^^2^ telescoping anastomosis,^3^ and the bronchial flap method to form the oblique of the distal bronchus.^4^^,^^5^

However, because we encountered a case of bronchopleural fistula the day after right sleeve pneumonectomy, we adjust the caliber difference in high-caliber mismatch cases by making a V-shaped incision in the cartilage of the proximal membranous transition area. Here we report that a V-shaped incision at the proximal cartilaginous section provides good results with respect to adjustment of high-caliber mismatch in bronchoplasty.

## Material and Methods

### Study Population

**Figure 1:**
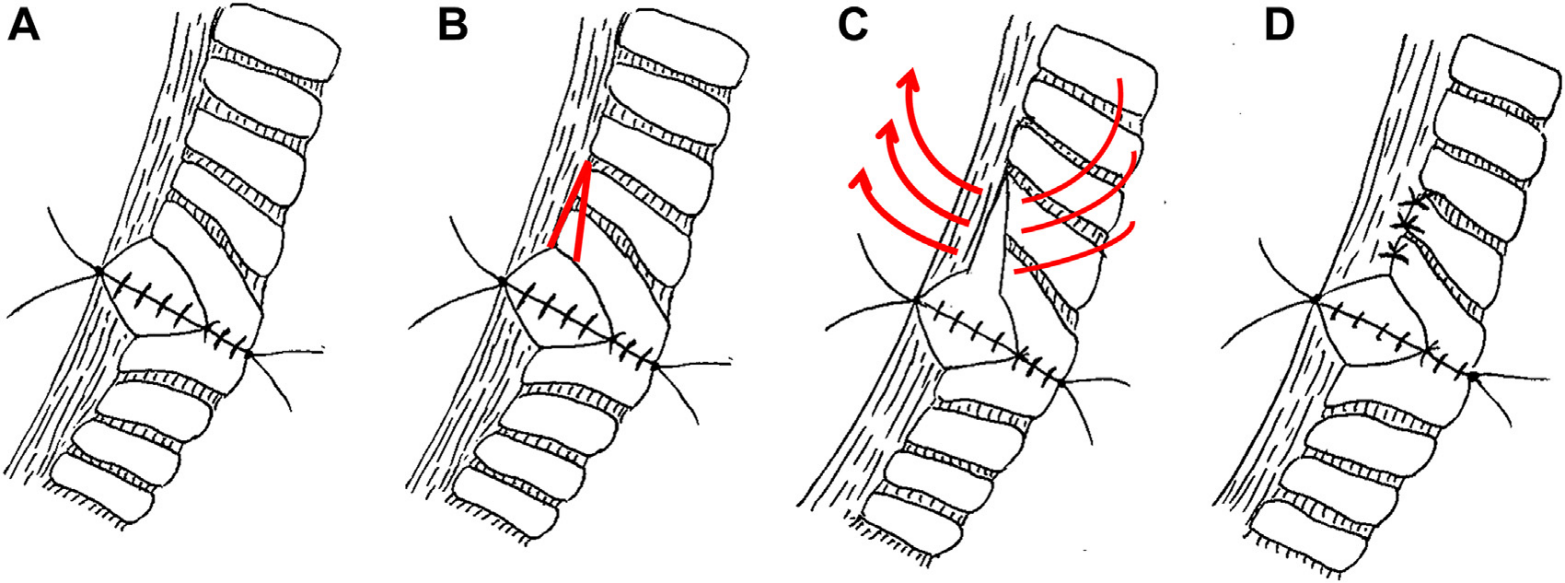
Adjustment technique for caliber mismatch using a proximal cartilaginous V-shaped incision. (A) Anastomotic site before proximal cartilaginous V-shaped incision. (B) A V-shaped incision is made in the proximal cartilage of the transitional part of the membranous portion. (C) Three interrupted 4-0 polydioxanone sutures are placed between the proximal cartilage and the membranous portion. (D) Findings at the completion of caliber adjustment at the proximal cartilage.

Between February 2008 and December 2022, 255 patients underwent tracheoplasty or bronchoplasty for non-small cell lung cancer at our hospital (Juntendo University School of Medicine, Tokyo, Japan). Of these patients, 12 (4.7%) underwent bronchoplasty with a proximal cartilage V-shaped incision for the adjustment of high-caliber mismatch (Figure 1). This retrospective review was performed under a waiver of authorization approved by the Institutional Review Board of the Juntendo University School of Medicine (IRB: E23-0253).

### Surgical Procedure, Postoperative Complications, and Follow-up Policy

Posterolateral thoracotomy was performed in all cases. Hilar and mediastinal lymph node dissections were performed in all patients. After the bronchial resection margin was sent for frozen section analysis, a continuous running suture (4-5 sutures) was applied to the deepest site of the bronchial stump by using 3-0 or 4-0 nonabsorbable monofilament sutures (Prolene, Ethicon Inc.). The rest of the anastomosis was performed using an interrupted suture with 3-0 or 4-0 nonabsorbable monofilament sutures, called a hybrid anastomosis.^6^ Before finishing the interrupted suture of the cartilaginous portion (Figure 1A), a V-shaped incision was made on the proximal side of the cartilage at the junction of the membranous portion (Figure 1B). The V-shaped incision was made only in the cartilaginous portion, not the membranous portion. The cartilage and membranous portion of the incision were adjusted using 3 interrupted 4-0 polydioxanone sutures (PDS, Ethicon Inc) (Figure 1C). Finally, interrupted sutures were placed in the membranous portion to complete the anastomosis (Figure 1D). As shown in Figure 1B, when making a V-shaped incision in the proximal cartilage, it is important to lengthen the long-axis incision relative to the short-axis incision in the cartilage. The anastomosis was covered with a pericardial fat pad where needed, especially in sleeve pneumonectomy, in patients with severe diabetes mellitus, or in patients receiving definitive chemoradiotherapy. After pulmonary resection, bronchoscopic examination was conducted on postoperative day 7 or before discharge. Routine bronchoscopic examinations were planned on postoperative days 14 and 28.

### Statistical Analysis

The χ^2^ test or the Fisher exact test was used to compare the frequencies of categoric variables. A complete case analysis was performed. A *P* value <.05 was considered statistically significant. All statistical analyses were performed using SPSS software version 26 (IBM Corp).

## Results

### Patient Characteristics

The overall clinicopathologic characteristics of the patients are summarized in the Supplemental Table. There were 12 patients, 11 (92%) of whom were men, and the median age was 66 years. The histologic diagnoses were adenocarcinoma in 2 patients (17%) and squamous cell carcinoma in 10 (83%) patients. Three patients (25%) underwent operation after definitive chemoradiotherapy. Right sleeve pneumonectomy, right sleeve upper lobectomy, type A extended-sleeve lobectomy, and type C extended-sleeve lobectomy were performed in 7 (58%), 3 (25%), 1 (8%), and 1 (8%) patients, respectively. Wrapping of the bronchial anastomosis was performed in 4 patients (33%) by using pericardial fat pads, and all of these patients had right sleeve pneumonectomy. Three of the 4 patients with a wrapped bronchial anastomosis were treated after definitive chemoradiotherapy, and the remaining patient had a case with high anastomotic tension. There was a significantly higher frequency of right sleeve pneumonectomy in the V-shaped cartilage incision group than in the non–V-shaped cartilage incision group (*P* < .01) (Table). The postoperative complications are shown in the Supplemental Table. No bronchial anastomosis-related complications were observed. One patient died 28 days after right sleeve pneumonectomy as a result of acute exacerbation of interstitial pneumonia.

**Table:** Comparison of Bronchoplasty Between V-shaped Cartilage Incision and Non–V-shaped Cartilage Incision

Variables	V-shaped Incision of the Cartilage, n (%) n = 12	Non–V-shaped Incision of the Cartilage, n (%) n = 243	*P* Value
Type of bronchoplasty			
Right sleeve pneumonectomy	7 (58)	24 (10)	<.01
Right sleeve upper lobectomy	3 (25)	73 (30)	.71
Type A extended-sleeve lobectomy	1 (8)	9 (4)	.42
Type C extended-sleeve lobectomy	1 (8)	19 (8)	.95

**Figure 2:**
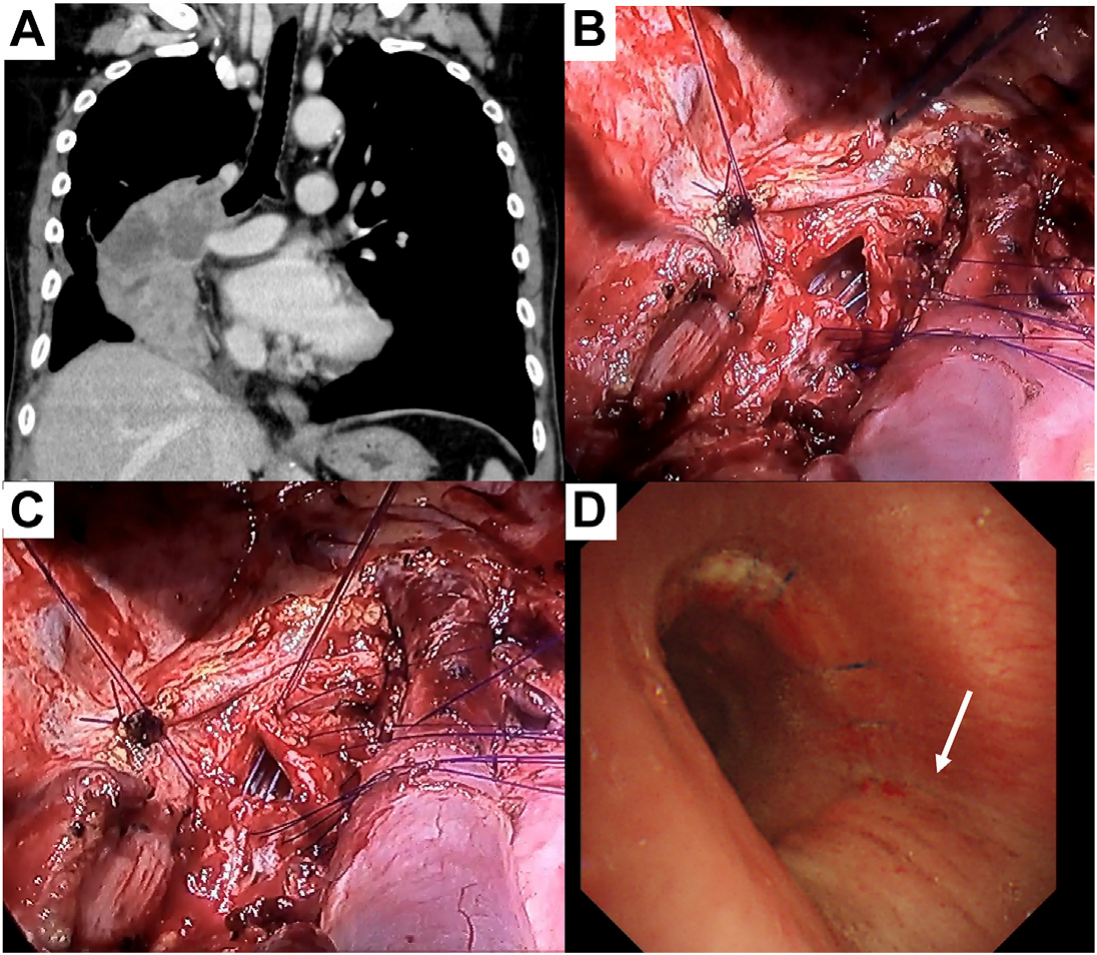
(A) Preoperative computed tomographic findings of the primary lung tumor in a 56-year-old man who underwent right sleeve pneumonectomy for lung squamous cell carcinoma (cT4 N2 M0) (B) Intraoperative findings of the anastomosis after a V-shaped proximal cartilaginous incision. (C) Anastomotic findings after completion of the proximal caliber adjustment. (D) Bronchoscopic findings of the anastomosis on the sixth postoperative day. Healing at the anastomotic site of the V-shaped incision (arrow) was good.

Figure 2 shows a case of caliber adjustment using a V-shaped incision in the proximal cartilage. A 56-year-old man underwent right sleeve pneumonectomy for lung squamous cell carcinoma (cT4 N2 M0) (Figure 2A). Intraoperatively, a caliber difference was detected between the stump of the trachea and the left main bronchus. Therefore, a V-shaped incision was made in the tracheal cartilage at the transition to the membranous part of the incision (Figure 2B). The cartilage and membranous part of the incision were sutured with 4-0 PDS (Figure 2C). On the sixth postoperative day, bronchoscopic findings of the anastomosis were good, including the suture at the V-shaped incision (Figure 2D). The postoperative course was uneventful, and the patient was discharged on the 14th postoperative day.

### Background of the Adjustment of Caliber Mismatch by Using a Proximal Cartilage V-shaped Incision

**Figure 3:**
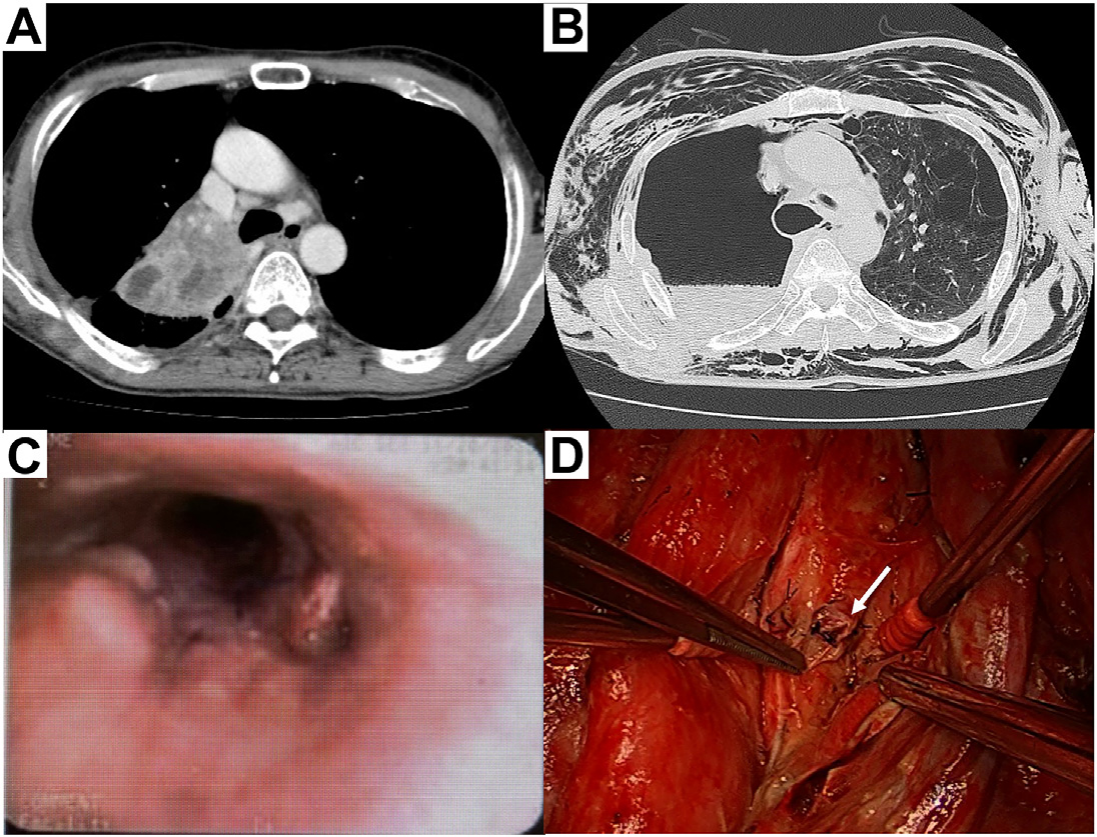
(A) Preoperative computed tomographic findings of a primary lung tumor in a 58-year-old man who underwent right sleeve pneumonectomy for lung squamous cell carcinoma (cT3 N1 M0). (B) Computed tomographic findings of subcutaneous emphysema exacerbation on the day after operation. (C) Bronchoscopic findings at the anastomosis between the trachea and the left main bronchus the day after operation. A fistula was observed between the right side of the cartilage and the membranous part of the anastomosis. (D) Intraoperative anastomotic findings during reoperation for the bronchial anastomotic fistula. One anastomotic thread on the right lateral wall of the cartilage at the anastomotic site (arrow) was broken.

A 58-year-old man underwent right sleeve pneumonectomy for lung squamous cell carcinoma (cT3 N1 M0) (Figure 3A). The operation was completed by covering the anastomosis with pericardial fat. However, on the day after operation, computed tomography revealed an increase in subcutaneous emphysema (Figure 3B), and bronchoscopy revealed a fistula in the cartilaginous area on the right side of the anastomosis (Figure 3C). Emergency reoperation was performed for bronchopleural fistula. After removing the pericardial fat pad at the anastomotic site, 1 anastomotic thread on the right lateral wall of the cartilage at the anastomotic site was broken (Figure 3D). We found that a bronchopleural fistula occurred because of high-caliber mismatch between the right lateral side of the trachea and the left main bronchus during the initial operation. A V-shaped incision was made in the proximal cartilaginous portion of the membranous transition area. An interrupted suture was placed between the trachea and the cartilaginous and membranous portions of the incision. Subsequently, reanastomosis was performed. The postoperative course was uneventful, with no anastomotic complications, and the patient was discharged on the 10th postoperative day.

## Comment

In this study, we report a technique for adjusting the high-caliber mismatch between the proximal and distal stumps. We made a V-shaped incision in the proximal cartilage of the transitional part of the membranous portion. The perioperative outcomes of our adjustment technique in all 12 patients were excellent, with no anastomotic complications.

During bronchoplasty, the problem of high-caliber mismatch is sometimes encountered. Large-caliber differences can lead to tension on the anastomosis and the risk of bronchopleural fistula. Therefore, it is important to use an appropriate anastomotic technique and adjust the stump to account for the high-caliber mismatch.

A previous study reported the safety and efficacy of telescoping anastomosis for high-caliber mismatch.^3^ Because our bronchoplasty policy is to anastomose from end to end, we did not use the telescopic method. Methods of enlarging the distal stump have been reported.^4^^,^^5^ However, this approach may disrupt the bronchial blood supply and cause bronchial anastomosis dehiscence secondary to ischemic changes. Regarding the proximal approach, reefing the membranous part of the bronchus^1^^,^^2^ and cutting the cartilage and membranous part followed by suturing^7^ have been reported. We did not use the membrane suture technique because the proximal membrane suture may pull the proximal cartilage of the anastomosis toward the membranous part, thereby causing tension in the cartilage.

Mori and colleagues^8^ reported a case of carinal cartilage suturing for high-caliber mismatch in right upper sleeve lobectomy. In this study, interrupted sutures were placed on the anterior wall of the carinal cartilage by pulling traction sutures on both stumps to reduce tension. These investigators reported that when proximal cartilages are sutured to each other, there is a concern that more tension may be placed on the proximal side of the reanastomosis. Therefore, we performed a V-shaped incision of the cartilage at the membranous transition and resutured the cartilage and membranous portion to relieve tension on the proximal side of the anastomosis. The key to releasing the tension is to make a V-shaped incision in the cartilage longitudinally relative to the short-axis incision length.

One of the problems in adjusting high-caliber mismatch in bronchoplasty is the difference in the shape of the proximal and distal sides of the bronchi. In right sleeve pneumonectomy or right upper sleeve lobectomy, both the proximal and distal bronchi have a horseshoe shape. In contrast, in left upper sleeve lobectomy, the proximal bronchus is horseshoe shaped, whereas the distal bronchus has a pavement-like shape (Supplemental Figure). If the distal bronchus is horseshoe shaped, the distal lumen does not widen during anastomosis. However, if the distal bronchus is pavement-like shaped, the presence of the intercondylar membrane enlarges the diameter of the distal bronchus, and this is advantageous for adjusting the caliber mismatch. We believe that the difference in shape between the proximal and distal stumps is one of the reasons that the V-shaped incision method for adjusting the caliber difference was significantly more frequently used in right sleeve pneumonectomy.

This study was limited by its single-center retrospective design and the small number of enrolled patients. Therefore, the statistical power may not be sufficiently high to guarantee reliable results. Nevertheless, the study provides new information on the adjustment of caliber mismatch by using a proximal cartilage V-shaped incision. Further research on our technique to adjust for caliber mismatch by using V-shaped proximal cartilage incisions in a larger group of patients is needed.

In conclusion, the proximal side of the cartilaginous V-shaped incision is a useful technique for adjusting high-caliber mismatch, especially in right sleeve pneumonectomy.

## Figures and Tables

**Supplemental Figure:**
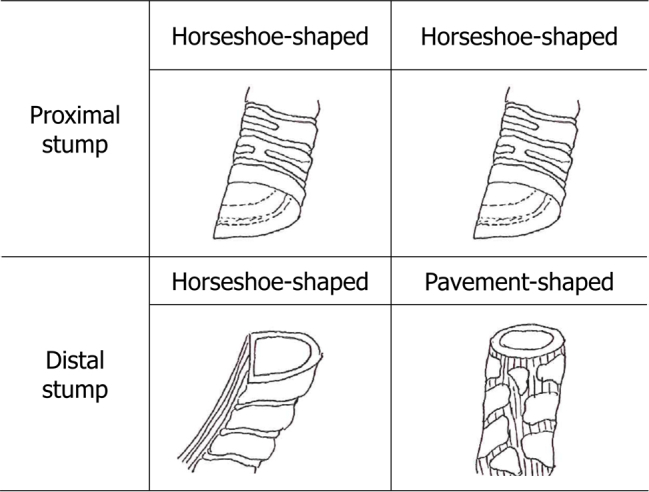


## References

[1] Watanabe Y., Shimizu J., Oda M. (1990). Results in 104 patients undergoing bronchoplastic procedures for bronchial lesions. Ann Thorac Surg.

[2] Berthet J.P., Paradela M., Jimenez M.J. (2013). Extended sleeve lobectomy: one more step toward avoiding pneumonectomy in centrally located lung cancer. Ann Thorac Surg.

[3] Hollaus P.H., Janakiev D., Pridun N.S. (2001). Telescope anastomosis in bronchial sleeve resections with high-caliber mismatch. Ann Thorac Surg.

[4] Ohata K., Zhang J., Ito S. (2013). Right lower lobe sleeve resection: bronchial flap to correct caliber disparity. Ann Thorac Surg.

[5] Hong T.H., Cho J.H., Shin S. (2018). Extended sleeve lobectomy for centrally located non-small-cell lung cancer: a 20-year single-centre experience. Eur J Cardiothorac Surg.

[6] Gomez-Caro A., Boada M., Reguart N. (2012). Sleeve lobectomy after induction chemoradiotherapy. Eur J Cardiothorac Surg.

[7] Grillo H.C. (1982). Carinal reconstruction. Ann Thorac Surg.

[8] Mori M., Takenaka M., Ichiki Y., Tanaka F. (2019). Simple carinal cartilage suture for high-caliber mismatch in right upper lobe sleeve lobectomy. Ann Thorac Surg.

