# Supplementary material for: V-Shaped Incision of the Proximal Cartilage for High-Caliber Mismatch in Bronchoplasty
Source: Ann Thorac Surg Short Rep. 2024 Apr 24;3(1):128–32. doi: 10.1016/j.atssr.2024.04.006 (PMC11910807; doi:10.1016/j.atssr.2024.04.006)
Supplement: Supplemental figure legend [file mmc1.docx]

**Supplemental figure legends**

*Supplemental Figure. The difference in the shape of the proximal and distal stump of the bronchi; horseshoe-shaped or pavement-shaped.*
