# Supplementary material for: V-Shaped Incision of the Proximal Cartilage for High-Caliber Mismatch in Bronchoplasty
Source: Ann Thorac Surg Short Rep. 2024 Apr 24;3(1):128–32. doi: 10.1016/j.atssr.2024.04.006 (PMC11910807; doi:10.1016/j.atssr.2024.04.006)
Supplement: Supplemental Table [file mmc2.docx]

*Supplemental Table. Characteristics of cases in which caliber mismatch was adjusted by the proximal side of cartilage V-shaped incision.*

| No | Age (y)  Sex | Preoperative  therapy | Type of  Operation | Vascular  reconstruction | Bronchial wrapping | Operative time  (min) | Blood loss  (ml) | Diagnosis  pStage | Complication |
| --- | --- | --- | --- | --- | --- | --- | --- | --- | --- |
| 1 | 58  Male | None | Right sleeve  pneumonectomy | None | Pericardial  fat pad | 267 | 270 | Sq  IIIB | Atrial fibrillation |
| 2 | 66  Male | CDDP+VNR  RT60Gy | Right sleeve  pneumonectomy | None | Pericardial  fat pad | 271 | 220 | Sq  IIIA | Atrial flutter |
| 3 | 67  Male | None | Type C extended  sleeve lobectomy | PA tangential  suture | None | 270 | 220 | Sq  IIA | Chylothorax |
| 4 | 57  Male | None | Right sleeve  pneumonectomy | None | None | 249 | 250 | Sq  IA | None |
| 5 | 71  Male | None | Right sleeve  upper lobectomy | None | None | 305 | 450 | Sq  IIIA | None |
| 6 | 66  Male | None | Right sleeve  upper lobectomy | None | None | 275 | 15 | Sq  IIIA | None |
| 7 | 76  Male | None | Right sleeve  upper lobectomy | None | None | 219 | 250 | Sq  IIB | Atelectasis |
| 8 | 56  Male | None | Right sleeve  pneumonectomy | None | None | 337 | 845 | Sq  IIIB | None |
| 9 | 70  Male | None | Right sleeve  pneumonectomy | None | None | 335 | 520 | Ad  IIIA | None |
| 10 | 63  Male | None | Type A extended  sleeve lobectomy | PA end-to-end  PV endo-to-end | None | 444 | 350 | Sq  IIIA | Prolonged air leak |
| 11 | 70  Male | CBDCA+PTX  RT60Gy | Right sleeve  pneumonectomy | None | Pericardial  fat pad | 220 | 230 | Ad  IVa | Interstitial pneumonia  acute exacerbation |
| 12 | 62  Female | CBDCA+PTX  RT60Gy  Durvalmab | Right sleeve  pneumonectomy | None | Pericardial  fat pad | 337 | 50 | Sq  IIIB | None |
| CDDP = cisplatin; VNR = vinorelbine; RT = radiation therapy; CBDCA = carboplatin; PTX = paclitaxel; PA = pulmonary artery; PV = pulmonary vein; Ad = adenocarcinoma; Sq = squamous cell carcinoma. | | | | | | | | | |
